# Destruction of polyelectrolyte microcapsules and release of FITC-dextran from them by the influence of sodium dodecyl sulfonate

**DOI:** 10.1038/s41598-022-08054-x

**Published:** 2022-03-07

**Authors:** Alexey V. Dubrovskii, Aleksandr L. Kim, Egor V. Musin, Sergey A. Tikhonenko

**Affiliations:** Institute of Theoretical and Experimental Biophysics, Russian Academy of Science, Institutskaya St., 3, 142290 Puschino, Moscow Region, Russia

**Keywords:** Drug delivery, Drug delivery, Supramolecular chemistry

## Abstract

Polyelectrolyte microcapsules can be applied as microcontainers for the delivery of a wide range of substances, and it is important to search for new methods for capsule destruction and releasing substances from them. In this work, we studied the possibility of using sodium dodecyl sulfonate (SDS) for the release of fluorescein isothiocyanate—dextran from six-layer microcapsules composed of PAH and PSS. It was shown that the presence of SDS in the medium, at a concentration of 3000 μg/ml, leads to the destruction of polyelectrolyte microcapsules and the release of the substance from them (54% of the amount of the encapsulated substance), while the main part of the FITC-dextran released during the first hours of incubation. At an SDS concentration of 100 μg/ml, the substance released is uniform and is 44% in 24 h. At SDS concentrations from 50 to 100 μg/ml, the process of destruction of microcapsules proceeds more slowly. At SDS concentrations from 10 to 50 μg/ml, microcapsules are not degraded.

## Introduction

In 1998, Donath et al. First demonstrated the application of LbL self-assembly technology to create polyelectrolyte microcapsules. Microcapsules were created by alternating deposition of oppositely charged polyelectrolytes on different particles due to their electrostatic attraction^1^. The variety of polyelectrolytes used makes it possible to encapsulate active substances of different nature and create microcontainers for specific tasks. Effective microencapsulation of drugs, proteins and enzymes, corrosion inhibitors and pesticides is becoming increasingly important for a wide range of applications in medicine, cosmetology, agriculture, etc.^2^.

One of the main advantages of polyelectrolyte microcapsules is a variety of methods for the controlled release of an encapsulated substance, due to the variability of the composition of the microcapsule shell^3^. Release from microcapsules is necessary in the development of targeted therapy, which is to deliver a drug to the target organ and its local release^4^. In addition, this technology is used to create: self-healing materials, for example, for the release of corrosion inhibitors^5,6^; a new type of pesticide^7^, for the gradual release of herbicides and reduce the burden on the environment; genomic editing tools^8^ etc.

The release of the encapsulated substance is usually achieved by disrupting the shell of the microcapsule. For example, microcapsules made from biodegradable polyelectrolytes of a protein nature are destroyed by proteolytic enzymes^9^. A change in the pH of the incubation medium can also be a trigger for degradation of the microcapsule envelope, depending on the pKa of polyelectrolytes^10^. The shell can also be destroyed due to the physicochemical features of the nanoparticles included in the shell^11–14^. For example, titanium oxide nanoparticles can disrupt the shell structure of microcapsules when exposed to ultraviolet radiation^15^. Even bacterial spores can be used to break down the membrane of the microcapsules^16^.

In this work, we propose a new approach to the destruction of the shell of polyelectrolyte microcapsules using a surfactant—Sodium dodecyl sulfonate (SDS). At the moment, surfactants are used to create microcapsules with certain properties^17^^,^^18^, for example, microcapsules with salt reception^18^. At the moment, there are no works in the literature on the release of substances using surfactants. But this technology can be used to create self-healing materials, for example, for the release of anticorrosive substances under the influence of SDS or in dental practice for the gradual release of drugs or antibiotics on the surface of dental implants, fillings and other elements.

## Materials and methods

### Materials

Polyelectrolytes polystyrenesulfonate sodium (PSS) and polyallylamine hydrochloride (PAH) with a molecular mass of 70 kDa, fluorescein isothiocyanate–dextran (150 kDa) Sigma (USA), Ethylenediaminetetraacetic acid disodium salt dihydrate (EDTA) purchased in Sigma (St. Louis, MS, USA), sodium dodecyl sulfonate (SDS), sodium carbonate, calcium chloride from “Reahim”.

### Preparation of CaCO_3_ Microspherulites

At stirring of 0.33 M Na_2_CO_3_, the 0.33 M CaCl_2_ containing 0,2 mg/ml of FITC-dextran was added. The stirring time was 30 s. The suspension was maintained until complete precipitation of the formed particles. The process of “ripening” of microspherolites was controlled with the help of a light microscope. Then, the supernatant was decanted, the precipitate was washed with water and used to prepare PMC. The microparticles were obtained with an average diameter of 4.5 ± 1 μm.

### Preparation of polyelectrolyte microcapsules

The polyelectrolyte microcapsules were obtained by layer-by-layer adsorbing the negatively or positively charged polyelectrolytes onto CaCO_3_ microspherulites, followed by dissolution of CaCO_3_. Layer-by-layer adsorption of PAH and PSS on the CaCO_3_ microspherulites surface was carried out in polyelectrolytes solutions (concentration 2 mg/mL + 0.5 M NaCl). After each adsorption the CaCO_3_ particles with adsorbed polyelectrolytes were triple washed with a 0.5 M NaCl solution, which was necessary to remove unabsorbed polymer molecules. The particles were separated from the supernatant by centrifugation. After applying the required number of layers, the carbonate kernels were dissolved in a 0.2 M EDTA solution for 12 h. The resulting capsules were washed three times with water to remove core decay products. The microcapsules were obtained with an average diameter of 4.5 ± 1 μm.

### Fluorescence microscopy

Images were taken using an «Axiovert 200 M Cell Observer» inverted fluorescence microscope with high-speed camera AxioCam HSM of Carl Zeiss company using 60 × /1.4 Oil objective. Diode laser 488 were used for excitation. Images were acquired at a resolution of 1388 × 1040 pixels.

### Incubation of PMC in SDS solution

The polyelectrolyte microcapsules with encapsulated FITC-dextran were incubated in SDS solution with concentrations 0, 10, 50, 100 and 3000 μg/ml with constant shaking on the vortex (500 rpm for the required time).

### Registration of the FITC-dextran release from polyelectrolyte microcapsules

After 0, 1, 3 and 24 h of incubation, a suspension of microcapsules was taken from the incubation solution and filtered using a membrane filter. Subsequently, the fluorescence intensity of the filtered solution was measured. Fluorescence spectra were recorded on an Infinite 200 Tecan instrument in a Black 96-Well Immuno Plates when excited with light at a wavelength of 480 nm.

## Results

### Destruction of polyelectrolyte microcapsules by an SDS

**Figure 1:**
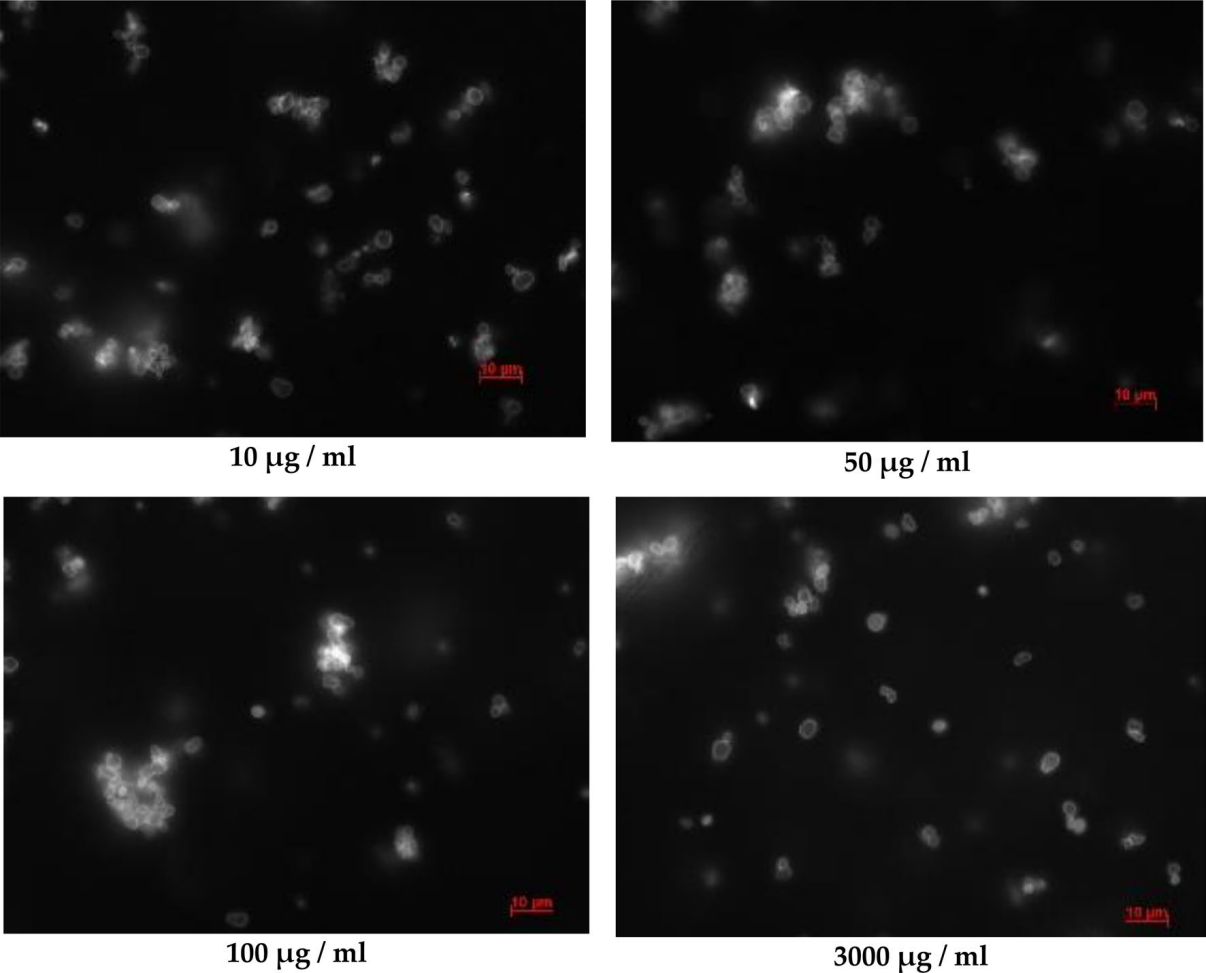
Image of polyelectrolyte microcapsules in SDS solution after 60 min of incubation(concentrations 10, 50, 100 and 3000 μg/ml).

The first stage of the research is to study the preservation of the integrity of polyelectrolyte microcapsules (PMC) over time in an SDS solution. For this, PMCs of composition (PSS/PAH) 3 containing FITC-labeled dextran were prepared. These microcapsules were incubated in SDS solutions with concentrations of 0, 10, 50, 100 and 3000 μg/ml and a sample of each concentration was photographed after 0, 10, 30, 60, 180 and 1440 (24 h) minutes of incubation. All obtained micrographs are presented in “Appendix” to this work. It was found that during 60 min of incubation, PMCs were not destroyed in the entire studied range of SDS concentrations. However, at concentrations of SDS from 10 to 100 μg/ml, aggregation of microcapsules was observed and the size of aggregates increased with increasing concentration of SDS (Fig. 1). At a concentration of sds of 3000 μg/ml, there was no aggregation of microcapsules, which may be due to the concentration of micelle formation of sds (CMC = 2300 μg/ml)^19^.

**Figure 2:**
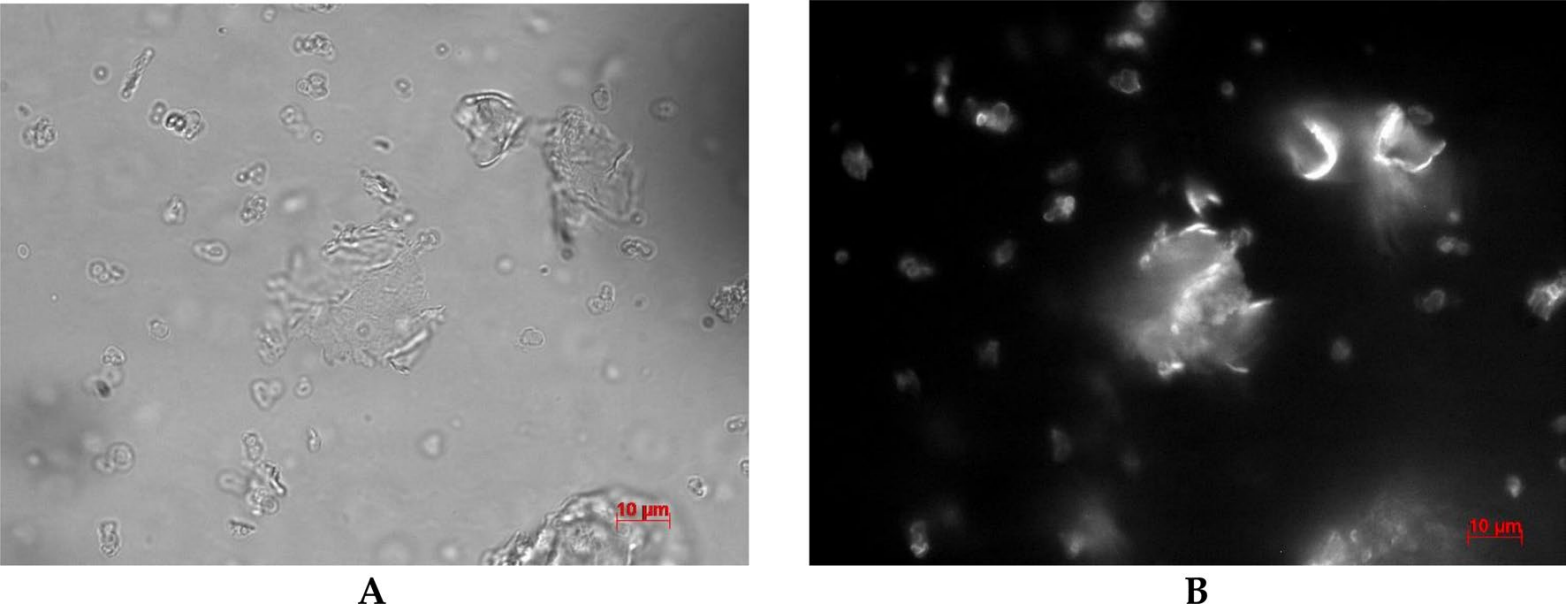
Image of polyelectrolyte microcapsules in 3000 μg/ml SDS solution after 180 min of incubation (**A**) light microscopy, (**B**) fluorescence microscopy).

After 180 min of incubation of the microcapsules, the destruction of the capsules in the SDS solution of 3000 μg/ml was observed (Fig. 2).

At this concentration, a large number of polyelectrolyte particles ranging in size from 6 to 20 μm with a complex irregular shape were observed, while undisturbed PMCs were also observed. At other concentrations, no changes were observed after 180 min of incubation.

**Figure 3:**
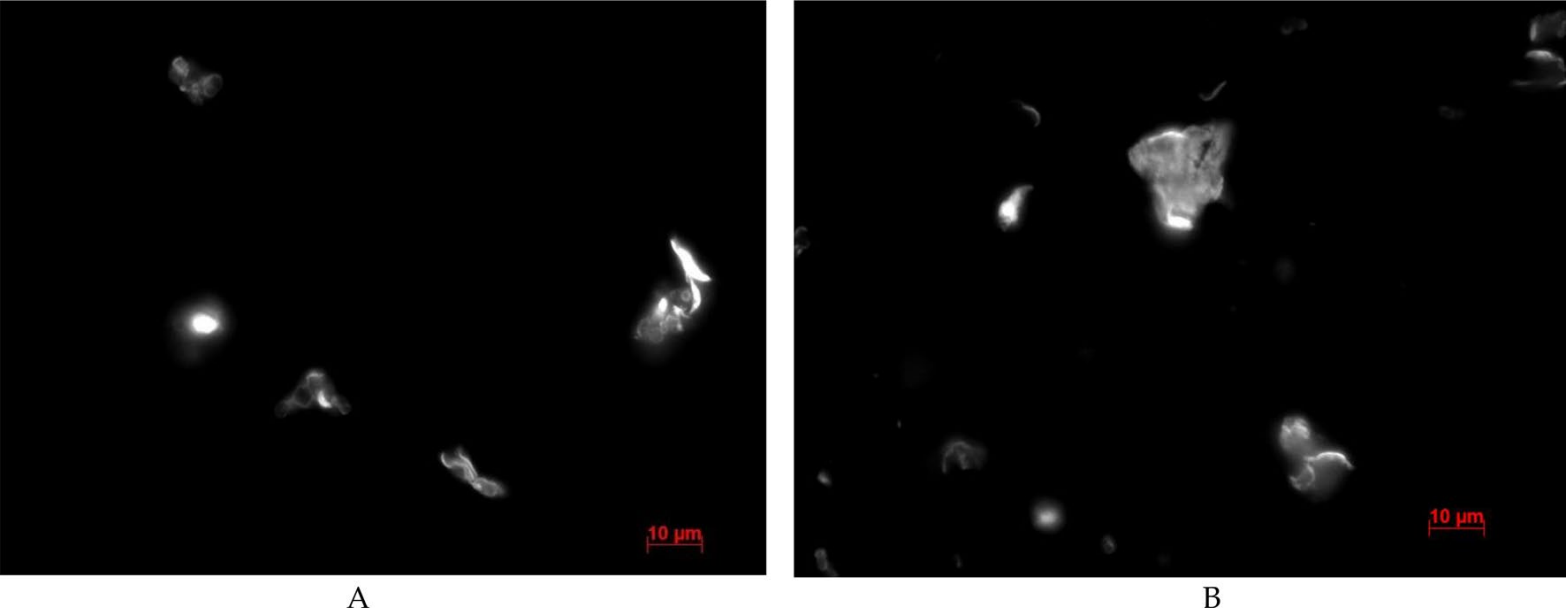
Image of polyelectrolyte microcapsules in 100 μg/ml (**A**) and 3000 μg/ml (**B**) SDS solution after 24 h of incubation.

After 24 h of incubation, degradation of the microcapsules was observed at SDS concentrations of 100 μg/ml and 3000 μg/ml (Fig. 3).

In the case of 100 μg/ml, both irregularly shaped polyelectrolyte particles and polyelectrolyte microcapsules are observed. In the case of 24-h incubation of PMC in a SDS solution with a concentration of 3000 μg/ml, only polyelectrolyte particles of irregular shape were observed.

**Figure 4:**
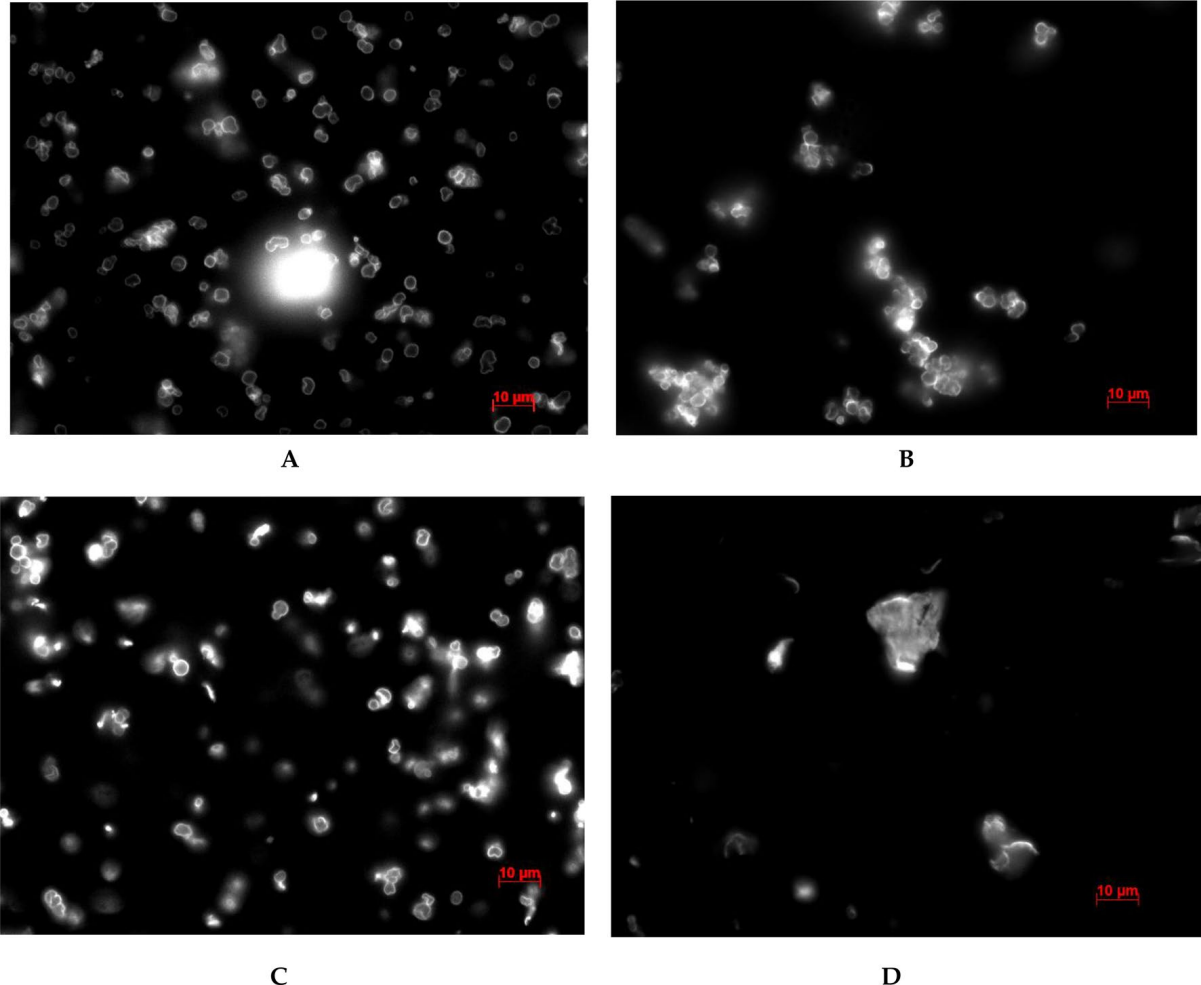
Image of polyelectrolyte microcapsules in water and 3000 μg/ml SDS solution before and after 24 h of incubation. (**A**) PMC in water solution before incubation. (**B**) PMC in 3000 μg/ml SDS solution before incubation. (**C**) PMC in water solution after 24 h incubation. (**D**) PMC in 3000 μg/ml SDS solution after 24 h incubation).

For clarity, Fig. 4 shows micrographs of microcapsules in a SDS solution of 3000 μg/ml and water before and after 24 h of incubation.

As can be seen from the figure, PMCs incubated in water are not destroyed during all 24 h. For 24 h of incubation in a SDS solution with a concentration of 3000 μg/ml, almost complete destruction of the capsules occurs; a large number of irregular particles, which are a polyelectrolyte complex, are observed in the solution. Thus, it can be concluded that the concentration of SDS 3000 μg/ml almost completely destroyed the PMC of the composition (PSS/PAH)_3_ within 24 h.

The cause of the destruction of the microcapsule may be the electrostatic interaction of SDS molecules with PAH amino groups. The work of A. Sharipova et al. describes the processes of complex formation between polyallylamine and sodium dodecyl sulfonate^20^. And the work of Eri Yoshida found out that an increase in the concentration of SDS in solution initiates the torsion of the polyelectrolyte and then the formation of a micellar aggregate, in the form of an SDS core coated with a PAH shell^21^. Based on the works described above, we put forward a hypothesis that SDS forms a complex with uncompensated site of PAH microcapsules, which leads to a weakening of the electrostatic interaction between subsequent bound regions of PSS and PAH and to the appereance of new uncompensated regions of polyelectrolytes. Further formation of PAH–SDS complexes leads to twisting and the formation of a micellar aggregate. The formation of the aggregate is accompanied by the release of the polyelectrolyte from the microcapsules, which with repetition, leads to their destruction. In addition, constant stirring of the solution speeds up this process.

### Release of FITC-labeled dextran from PMC

**Figure 5:**
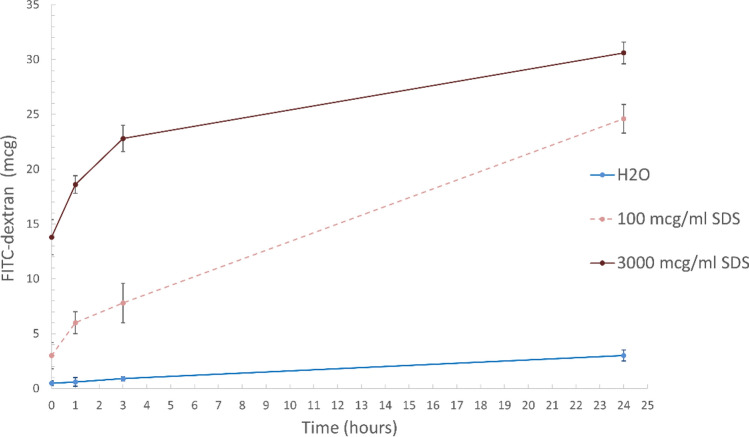
The release of FITC-dextran over time from polyelectrolyte microcapsules in SDS solution (100 and 3000 μg/ml).

The next stage of our research was to study the release of encapsulated FITC-labeled dextran from PMC due to the effect of SDS. We used an SDS solution with concentrations of 0, 100, and 3000 μg/ml, since at these concentrations, micrographs showed destruction of the PMC shape after 24 h of incubation. The change over time in the amount of FITC-labeled dextran is shown in Fig. 5.

The figure shows that at the moment of adding capsules to the SDS solution (3000 μg/ml), a significant increase in the amount of FITC-dextran in the incubation solution is observed. In the case of an SDS concentration of 100 μg/ml, a similar effect is observed but much less expressed. Subsequently, there is a gradual release of FITC-dextran into the incubation solution in all samples. The release of FITC-dextran in trace amounts is observed, when PMC is incubated in water, a similar result for capsules of the composition (PSS/PAH)_3_ was described in other works (1, 2, 3). The highest release of FITC-labeled dextran is observed at an SDS concentration of 3000 μg/ml, and the main release is observed in the first 3 h of incubation. The release of FITC-dextran in an SDS solution with a concentration of 100 μg/ml is more uniform. Differences in the release of dextran at concentrations of 100 μg/ml and 3000 μg/ml are observed due to the fact that in a higher concentration of SDS microcapsules are destroyed faster (after 3 h), which leads to a faster release of the substance in the first hours of incubation. The total release of dextran in 24 h at an SDS concentration of 3000 μg/ml is 54% of the amount of the encapsulated substance; at an SDS concentration of 100 μg/ml, the dextran release is 44%.

## Conclusions

The work demonstrated the destruction of the PMC using SDS. It was found that in a high concentration SDS solution (3000 μg/ml), microcapsules begin to be destroyed after 3 h of incubation, and almost destroyed in 24 h. A lower concentration of SDS (100 μg/ml) also leads to the destruction of capsules, however, this process is more prolonged in time and not so expressed. At SDS concentrations ranging from 10 to 50 μg/ml, the destruction of microcapsules was not observed. The study of the release of FITC-labeled dextran from microcapsules showed that at a SDS concentration of 3000 μg/ml, the main release occurs during the first hours of incubation and in total for 24 h is 54% of the amount of the encapsulated substance. At an SDS concentration of 100 μg/ml, the dextran release occurs evenly and is 44% in 24 h. These data correlate well with the results of an experiment to study the destruction of microcapsules. The cause of the destruction of the microcapsule may be the electrostatic interaction of SDS molecules with uncompensated site of PAH microcapsules, which leads to the destruction of the PMC.

## Appendix

The images of polyelectrolyte microcapsules in SDS solution (concentration 0, 10, 50, 100 and 3000 μg/ml) over time (after 0, 10, 30, 60, 180 and 1440 (24 h) minutes).

## References

[1] Donath E, Sukhorukov GB, Caruso F, Davis SA, Möhwald H (1998). Novel hollow polymer shells by colloid-templated assembly of polyelectrolytes. Angew. Chemie Int. Ed..

[2] Wang C, Ye S, Sun Q, He C, Ye W, Liu X, Tong Z (2008). Microcapsules for controlled release fabricated via layer-by-layer self-assembly of polyelectrolytes. J. Exp. Nanosci..

[3] De Geest BG, Sanders NN, Sukhorukov GB, Demeester J, De Smedt SC (2007). Release mechanisms for polyelectrolyte capsules. Chem. Soc. Rev..

[4] Johnston APR, Cortez C, Angelatos AS, Caruso F (2006). Layer-by-layer engineered capsules and their applications. Curr. Opin. Colloid Interface Sci..

[5] Zheludkevich ML, Shchukin DG, Yasakau KA, Möhwald H, Ferreira MGS (2007). Anticorrosion coatings with self-healing effect based on nanocontainers impregnated with corrosion inhibitor. Chem. Mater..

[6] Kartsonakis IA, Danilidis IL, Pappas GS, Kordas GC (2010). Encapsulation and release of corrosion inhibitors into titania nanocontainers. J. Nanosci. Nanotechnol..

[7] Wang X, Zhao J (2013). Encapsulation of the herbicide picloram by using polyelectrolyte biopolymers as layer-by-layer materials. J. Agric. Food Chem..

[8] Santos JL, Nouri A, Fernandes T, Rodrigues J, Tomás H (2012). Gene delivery using biodegradable polyelectrolyte microcapsules prepared through the layer-by-layer technique. Biotechnol. Prog..

[9] Borodina TN, Rumsh LD, Kunizhev SM, Sukhorukov GB, Vorozhtsov GN, Feldman BM, Markvicheva EA (2008). Polyelectrolyte microcapsules as the systems for delivery of biologically active substances. Biochem. Suppl. Ser. B Biomed. Chem..

[10] Sukhorukov GB, Antipov AA, Voigt A, Donath E, Mhwald H (2001). pH-controlled macromolecule encapsulation in and release from polyelectrolyte multilayer nanocapsules. Macromol. Rapid Commun..

[11] Skirtach AG, Antipov AA, Shchukin DG, Sukhorukov GB (2004). Remote activation of capsules containing ag nanoparticles and ir dye by laser light. Langmuir.

[12] Bukreeva T, Orlova A, Sulyanov S, Grigoriev Y, Dorovatovskii P (2011). A New Approach to Modification of Polyelectrolyte Capsule Shells by Magnetite Nanoparticles. Crystallogr. Rep..

[13] Marchenko IV, Plotnikov GS, Baranov AN, Saletskii AM, Bukreeva TV (2010). Formation and destruction of polyelectrolyte microcapsules modified by rhodamine 6G. J. Surf. Investig. X-ray Synchrotron Neutron Tech..

[14] Gulyaev YV, Cherepenin VA, Vdovin VA, Taranov IV, Sukhorukov GB, Gorin DA, Khomutov GB (2015). Decapsulation of polyelectrolyte nanocomposite microcapsules by pulsed microwave effect. J. Commun. Technol. Electron..

[15] Demina PA, Degtyareva EV, Kuzmicheva GM, Bukreeva TV (2014). Polyelectrolyte microcapsules modified with nanosized titanium dioxide for targeted drug delivery. Chem. Technol. Inorg. Mater..

[16] Musin EV, Kim AL, Dubrovskii AV, Kudryashova EB, Tikhonenko SA (2019). Decapsulation of dextran by destruction of polyelectrolyte microcapsule nanoscale shell by bacillus subtilis bacteria. Nanomaterials.

[17] Sharipova A, Aidarova S, Mutaliyeva B, Babayev A, Issakhov M, Issayeva A, Madybekova G, Grigoriev D, Miller R (2017). The Use of polymer and surfactants for the microencapsulation and emulsion stabilization. Colloids Interfaces.

[18] Duan G, Haase MF, Stebe KJ, Lee D (2018). One-Step generation of salt-responsive polyelectrolyte microcapsules via surfactant-organized nanoscale interfacial complexation in emulsions (SO NICE). Langmuir.

[19] Hammouda B (2013). Temperature effect on the nanostructureof SDS micelles in water. J. Res. Natl. Inst. Stand. Technol..

[20] Sharipova A, Aidarova S, Fainerman VB, Stocco A, Cernoch P, Miller R (2011). Dynamics of adsorption of polyallylamine hydrochloride/sodium dodecyl sulphate at water/air and water/hexane interfaces. Colloids Surfaces A Physicochem. Eng. Asp..

[21] Yoshida E (2010). Self-assembly of poly(allylamine hydrochloride) through electrostatic interaction with sodium dodecyl sulfate. Colloid Polym. Sci..

